# Foveal Remodeling of Retinal Microvasculature in Parkinson’s Disease

**DOI:** 10.3389/fnins.2021.708700

**Published:** 2021-07-12

**Authors:** Ane Murueta-Goyena, Maitane Barrenechea, Asier Erramuzpe, Sara Teijeira-Portas, Marta Pengo, Unai Ayala, David Romero-Bascones, Marian Acera, Rocío Del Pino, Juan Carlos Gómez-Esteban, Iñigo Gabilondo

**Affiliations:** ^1^Neurodegenerative Diseases Group, Biocruces Bizkaia Health Research Institute, Barakaldo, Spain; ^2^Department of Preventive Medicine and Public Health, University of the Basque Country (UPV/EHU), Leioa, Spain; ^3^Biomedical Engineering Department, Faculty of Engineering, Mondragon Unibertsitatea (MU-ENG), Mondragon, Spain; ^4^Department of Molecular and Translational Medicine, University of Brescia, Brescia, Italy; ^5^Neurology Department, Cruces University Hospital, Barakaldo, Spain; ^6^Department of Neurosciences, University of the Basque Country (UPV/EHU), Leioa, Spain; ^7^Ikerbasque: The Basque Foundation for Science, Bilbao, Spain

**Keywords:** neurodegeneration, angiography, capillary, density, Parkinson’s disease, retina, optical coherence tomography, biomarker

## Abstract

**Background:**

Retinal microvascular alterations have been previously described in Parkinson’s disease (PD) patients using optical coherence tomography angiography (OCT-A). However, an extensive description of retinal vascular morphological features, their association with PD-related clinical variables and their potential use as diagnostic biomarkers has not been explored.

**Methods:**

We performed a cross-sectional study including 49 PD patients (87 eyes) and 40 controls (73 eyes). Retinal microvasculature was evaluated with Spectralis OCT-A and cognitive status with Montreal Cognitive Assessment. Unified PD Rating Scale and disease duration were recorded in patients. We extracted microvascular parameters from superficial and deep vascular plexuses of the macula, including the area and circularity of foveal avascular zone (FAZ), skeleton density, perfusion density, vessel perimeter index, vessel mean diameter, fractal dimension (FD) and lacunarity using Python and MATLAB. We compared the microvascular parameters between groups and explored their association with thickness of macular layers and clinical outcomes. Data were analyzed with General Estimating Equations (GEE) and adjusted for age, sex, and hypertension. Logistic regression GEE models were fitted to predict diagnosis of PD versus controls from microvascular, demographic, and clinical data. The discrimination ability of models was tested with receiver operating characteristic curves.

**Results:**

FAZ area was significantly smaller in patients compared to controls in superficial and deep plexuses, whereas perfusion density, skeleton density, FD and lacunarity of capillaries were increased in the foveal zone of PD. In the parafovea, microvascular parameters of superficial plexus were associated with ganglion cell-inner plexiform layer thickness, but this was mainly driven by PD with mild cognitive impairment. No such associations were observed in controls. FAZ area was negatively associated with cognition in PD (non-adjusted models). Foveal lacunarity, combined with demographic and clinical confounding factors, yielded an outstanding diagnostic accuracy for discriminating PD patients from controls.

**Conclusion:**

Parkinson’s disease patients displayed foveal microvascular alterations causing an enlargement of the vascular bed surrounding FAZ. Parafoveal microvascular alterations were less pronounced but were related to inner retinal layer thinning. Retinal microvascular abnormalities helped discriminating PD from controls. All this supports OCT-A as a potential non-invasive biomarker to reveal vascular pathophysiology and improve diagnostic accuracy in PD.

## Introduction

Parkinson’s disease (PD) is a progressive neurodegenerative disease characterized by motor impairment, including rest tremor, muscle rigidity, bradykinesia, and postural imbalance. The main hallmark of PD is the accumulation of anomalous α-synuclein deposits within neuronal cytoplasm, presumably resulting in profound loss of neurons, mainly of dopaminergic neurons (Lotharius and Brundin, 2002). The pathological features of PD have also been observed in postmortem retinas (Ortuno-Lizaran et al., 2018), and several *in vivo* cross-sectional studies have reported reduced retinal thickness in PD by means of optical coherence tomography (OCT) (Chrysou et al., 2019). Retinal atrophy seems to be specific to the inner retinal layers, concretely, to macular ganglion cell-inner plexiform complex (GCIPL) around the fovea (Murueta-Goyena et al., 2019), where the largest amount of retinal dopaminergic cells is found (Ortuño-Lizarán et al., 2020). The GCIPL thinning is significantly more pronounced in PD patients over time compared to controls (Murueta-Goyena et al., 2021), but it is present from prodromal stages (Lee et al., 2019a,b), suggesting that an early but active neurodegeneration takes place in PD retina (Murueta-Goyena et al., 2021).

Previous publications have indicated that, in addition to neurodegeneration, the vascular component might be a key contributing factor to the pathogenesis and progression of PD (Bradaric et al., 2012; Guan et al., 2013; Yang et al., 2015). In fact, brain autopsies of PD patients have revealed capillary disruption (Guan et al., 2013), angiogenesis (Bradaric et al., 2012), and small vessel degeneration in substantia nigra, middle frontal cortex and brainstem nuclei (Yang et al., 2015). It has been suggested that retinal vasculature shows similarities with cerebral microcirculation and can be therefore used as a surrogate marker of cerebral microvascular pathology (Patton et al., 2005). Within the retina, blood flow to inner retinal layers comes from capillaries derived from the central retinal artery, whereas outer retinal layers are supplied by choroidal vasculature. Recent advances in OCT technology allow the visualization of retinal vasculature using non-invasive, depth-selective, and high-resolution images. OCT angiography (OCT-A) detects blood flow down to the capillary level by measuring changes in OCT signal in consecutive cross-sectional images taken at the same location and allows a three-dimensional mapping of retinal microvasculature. Studying the morphometric variations of capillary networks in PD might provide key information about the regional neuronal structure, and the basis for investigating retinal vascular morphological features as potential biomarkers of cerebral microcirculation in PD. Although to date few studies have explored retinal vascular alterations in PD using OCT-A, the observations so far support the view that retinal vascular alterations are present in PD (Kromer et al., 2016; Kwapong et al., 2018; Rascunà et al., 2020; Shi et al., 2020; Zou et al., 2020; Robbins et al., 2021).

On the other hand, the relationship between cerebral small vessel disease and cognitive decline is well-established (Zanon Zotin et al., 2021). In PD, cognitive impairment is present in 15 to 40% of patients at diagnosis or early stages of the disease (Aarsland et al., 2009; Pfeiffer et al., 2014), and about 80% of PD patients will progress to dementia, but the rate of disease progression is not uniform across patients (Aarsland et al., 2017). Recent evidence shows that subjects with mild cognitive impairment (MCI) display retinal vascular network impairment (Chua et al., 2020; Criscuolo et al., 2020; Shin et al., 2021). Similarly, it seems that PD patients with GCIPL atrophy might constitute a clinical endophenotype with more pronounced cognitive impairment and worse prognosis (Murueta-Goyena et al., 2019, 2021). However, the relationship between the cognitive status, retinal microvascular parameters and retinal layer thicknesses has not been fully explored in PD patients.

In this study, we aimed to extensively describe retinal vascular morphometric parameters in PD patients using high-resolution Spectralis OCT-A images, in order to verify the presence of microvascular abnormalities in PD compared to controls or specific alterations in PD-MCI compared to PD patients with normal cognition. We also evaluated the association of retinal microvascular parameters with retinal thickness measurements and disease-related clinical variables. Finally, we assessed the diagnostic accuracy of retinal microvascular parameters alone or in combination with the thickness of retinal layers to differentiate PD patients from controls.

## Materials and Methods

### Design and Participants

55 patients with Parkinson’s disease (105 eyes) and 48 controls (95 eyes) were initially recruited for the present cross-sectional study from June 2020 to March 2021. We included individuals aged 40 years or older. PD patients were recruited through the outpatient neurology department at Cruces University Hospital and fulfilled PD Parkinson’s UK Brain Bank criteria for the diagnosis of PD before enrollment. Demographic data, disease onset, disease severity and type and dosage of dopaminergic treatment were collected. One experienced neurologist in the field of movement disorders recorded disease onset, Unified Parkinson’s Disease Rating Scale (UPDRS) score, and calculated Levodopa Equivalent Daily Dose (LEDD). All patients were studied in an on-state of medication. Control individuals without PD or a history or symptoms of other neurological conditions were also enrolled in the study. Montreal Cognitive Assessment (MoCA) was administered to all participants to evaluate general cognition. A cutoff of 24 was established for determining MCI in this Spanish population (Milani et al., 2018).

All participants completed a comprehensive questionnaire on current comorbidities to check for the following systemic exclusion criteria: severe smoking (>20 cigarettes/day), heavy alcohol use (>4 drinks/day for men or >3 drinks/day for women), diagnosis of any type or grade of diabetes, uncontrolled or resistant elevated blood pressure, history of consumption of drugs or medications known to induce retinal toxicity or cognitive impairment, chronic inflammatory systemic diseases (e.g., lupus erythematosus, sarcoid, Bechet disease), history of brain trauma or central nervous system diseases different from PD. Participants with well-controlled hypertension without complications were included in the study.

PD patients and controls underwent a complete ophthalmologic examination including pupillary reflexes, refraction, visual acuity, color discrimination, slit lamp examination, and spectral domain OCT. Spherical equivalent refractive error above 4.00 diopters or more than 3.00 diopters of astigmatism or any ocular or systemic pathological condition, except PD, influencing retinal OCT measures were considered exclusion criteria. OCT-A images with visually identifiable motion artifacts or incomplete acquisitions were excluded from the analyses.

The study protocol was approved by the regional Basque Clinical Research Ethics Committee. All participants gave written informed consent prior to their participation in the study, in accordance with the tenets of the Declaration of Helsinki.

### Spectral Domain Optical Coherence Tomography (OCT)

Macular retinal thickness was assessed using the Spectralis spectral-domain OCT system (HRA2 Acquisition Module version 6.16.6.0, Heidelberg Engineering, Heidelberg, Germany). Macular volumetric scans consisted of 25 single horizontal axial scans (B-scans) covering a 20° × 20° area, with 512 A-scans per B-scan and 49 frames averaged per B-scan. Layer segmentation of the OCT data was performed with the built-in software. All OCT images fulfilled quality control criteria from OSCAR-IB consensus (Tewarie et al., 2012), accounting for Obvious problems (O), poor Signal strength (S), Centration of scan (C), Algorithm failure (A), Retinal pathology other than PD-related (R), Illumination (I), and Beam placement (B).

Macular volumetric scans were exported in raw format (^∗^.vol) and imported into MATLAB 2018b and 2019b (Mathworks, Natick, MA, United States) using the openVolFast.m function of the AURA tools software (Lang et al., 2013). The central point of the macula was determined as the point of minimum thickness after smoothing the thickness map with a circular kernel of 0.05 mm radius (foveaFinder.m function of AURA tools). The thickness values derived from the acquired raster pattern were resampled to a regular grid using cubic interpolation and 0.02 mm spacing between adjacent points. Then, the average thickness within the foveal zone (central 1-mm diameter disc) and parafoveal area (2.5-mm diameter ring adjacent to the foveal zone) were computed by averaging the point-by-point thicknesses in each sector.

**FIGURE 1:**
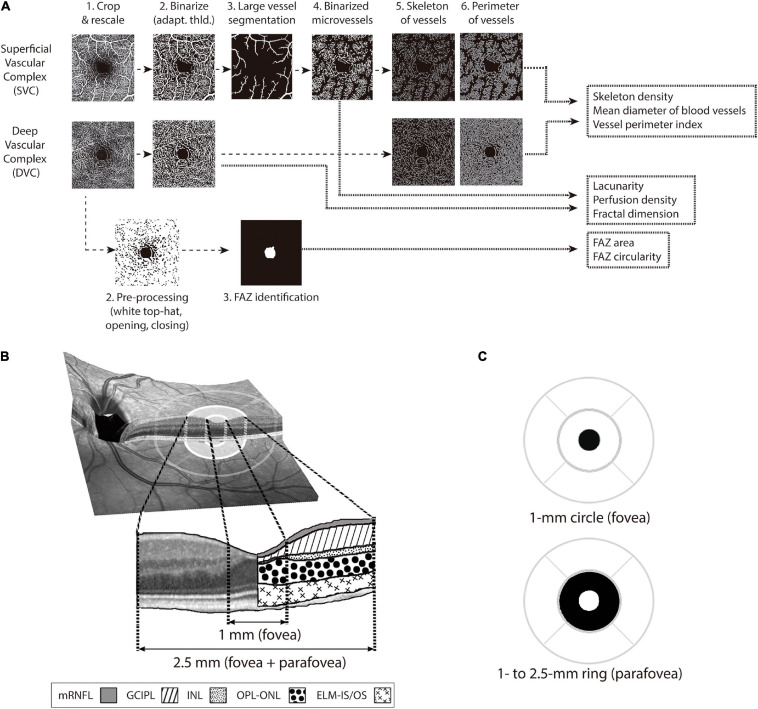
OCT-A image processing pipeline and evaluated retinal layers and regions for OCT and OCT-A images. The analysis protocol of the OCT-A images **(A)** was different for the superficial vascular complex (SVC) and for the deep vascular complex (DVC) (see “Materials and Methods” for more details). Both SVC and DVC images were cropped & rescaled (1) and binarized with an adaptive threshold (“adapt. thld.”) (2). In SVC images, after being binarized, the large vessels were segmented (3) and the microvessels were binarized (4). With binarized SVC microvessel and binarized DVC images, images for vessel skeleton (5) and perimeters (6) were obtained. From the vessel skeleton images, the “skeleton density” was quantified, from vessel perimeter images the “vessel perimeter index” was computed and from the combination of vessel skeleton and perimeter images, the “mean diameter of blood vessels” was computed. From the binarized images of SVC and DVC, the parameters “Lacunarity,” “Perfusion density,” and “Fractal dimension” were calculated. Finally, the cropped & rescaled DVC images were pre-processed with white top-hat and opening/closing to segment the foveal avascular zone (FAZ) mask, from which “FAZ area” and “FAZ circularity” were computed. The layers of the retina that were segmented from the OCT images **(B)** were macular retinal nerve fiber layer (mRNFL), ganglion cell-inner plexiform layer complex (GCIPL), inner nuclear layer (INL), outer plexiform layer, Henle fibers, and outernuclear layer complex (OPL-ONL) and the complex including external limiting membrane and internal andouter segments of photoreceptors (ELM-IS/OS). The mean thickness of the mentioned layers was obtained for two macular regions **(B,C)**: the foveal region (central circle of 1 mm in diameter) and the parafoveal ring (centered in the fovea and delimited by circles with an inner diameter of 1-mm and outer diameter of 2.5 mm). For both regions, the mean thicknesses of the mentioned retinal layers and the described OCT-A parameters were calculated.

The foveal and parafoveal thicknesses were calculated for the following layer complexes: total retinal thickness (Retina), macular nerve fiber layer (mRNFL), ganglion cell-inner plexiform complex (GCIPL), inner nuclear layer (INL), outer plexiform-Henle fiber-outer nuclear layer (OPL-ONL), and the complex including external limiting membrane and photoreceptor inner and outer segments (ELM-IS/OS) (Figure 1).

### Spectral Domain Optical Coherence Tomography Angiography (OCT-A)

High-resolution acquisition was performed with Spectralis OCT Angiography Module (Heidelberg Engineering, Germany) which offers a lateral resolution of 5.7 μm and an axial resolution of 3.9 μm per pixel, using a scanning area of 10° × 10° and 512 A-scans per B-scan. TruTrack Active Eye Tracking was used to avoid motion artifacts. Superficial and deep vascular plexus complexes of the macula were investigated [superficial vascular complex (SVC) located between the ganglion cell layer and the inner mid-part of inner plexiform layer and deep vascular complex (DVC) located between the outer mid-part of inner plexiform layer and outer plexiform layer]. *En face* images were exported from Spectralis and stored as 768 × 768 pixels jpeg images.

### OCT-A Image Processing

MATLAB 2018b and 2019b and Python (v3.8.5) were used to develop image analysis and parameter extraction algorithms. For vascular and foveal feature extraction, *en face* OCT-A images were first cropped to remove the SLO funduscopic image of the periphery. Then, OCT-A images were scaled using X and Y axis scaling parameters from Spectralis OCT-A to obtain images of 1:1 pixel:μm correspondence. We defined the foveal zone as the central 1-mm disc, and the parafovea as the ring surrounding the foveal zone, with an outer diameter of 2.5 mm. The center of the inner 1-mm diameter circle and outer 2.5 mm diameter ring was the centroid of the FAZ in DVC, i.e., the same center location was used in SVC and DVC for calculating microvascular parameters in different concentric regions (Figure 1).

For microvascular parameter extraction, OCT-A images were first enhanced using median filtering and later processed with a top-hat filter to improve the contrast in the image. The images were binarized using adaptive thresholding. In SVC, a separate binarization algorithm was applied based on Otsu’s threshold to segment large blood vessels. By subtracting large vessel segmentation to the binarized OCT-A images, we obtained SVC microvasculature. As the OCT-A detects blood flow down to the capillary level by measuring the changes in OCT signal in consecutive cross-sectional images, the whiteness of the binarized images reflects the probability of perfusion. This allowed us to calculate microvascular perfusion density as the ratio between the number of white pixels of the binarized image and the total number of pixels in the region of interest. The binarized images of the microvasculature were further processed to extract the skeleton of the vasculature using a built-in function in MATLAB. Furthermore, a Canny edge detector was implemented to detect the borders of the vasculature. From these images, we computed the following microvascular parameters: skeleton density as the number of pixels of the skeleton divided by the number of pixels in the region, the vessel perimeter index (Alam et al., 2017), mean vessel diameter (Alam et al., 2017), the fractal dimension (FD) estimated with Hausdorff (Box-counting) method (Harrar and Hamami, 2007) and lacunarity with gliding box method (Tolle et al., 2008). The lacunarity parameter used herein refers to the calculation of lacunarity using a box-size of 512 pixels, which represents a relative box-size of 0.1741 with respect to the rescaled binarized image. These parameters were extracted in the foveal zone and the parafovea. FD, lacunarity and perfusion density were dimensionless, skeleton density and vessel perimeter index were measured in 1/mm (length per unit of area) and mean vessel diameter in μm.

For foveal avascular zone (FAZ) parameter extraction, FAZ was first segmented using a parameterized version of Díaz et al. (2019). The process consisted of a top hat transform for image enhancement, Canny edge detection processing (including a Gaussian filter), and the application of opening and closing morphological operations to remove noise and fill holes. The FAZ area (mm^2^) was measured, and the circularity was calculated with the following formula: 4π (area/perimeter^2^).

### Statistical Analysis

Statistical analysis was done in R (version 3.6.1) and RStudio (version 1.2.1335). Group differences of demographic categorical variables were tested using Chi square test. Quantitative variables were described using mean and standard deviation. Normality of data was visually inspected and tested with Shapiro-Wilks. Group comparisons of normally distributed variables were done with *T*-test and non-normally distributed data assessed with Mann Whitney *U*-test. The analyses of OCT and OCT-A parameters were conducted using generalized estimating equation (GEE) models with an exchangeable working correlation structure to account for correlation between the two eyes from a single participant. Effect sizes were calculated with Cohen’s d. To test the diagnostic ability of OCT-A parameters alone or in combination with demographic or retinal thickness variables, we fitted logistic GEE models and their predictive ability was tested in ROC curves, using fitted values as predictors. For this, we first fitted the null model including age, sex, and hypertension as *a priori* confounders, and then added retinal variables for the full model. The differences in goodness-of-fit between models were tested with Wald test. All GEE analyses were performed with geepack package and ROC curves calculated with pROC package. *p-values* lower than 0.05 were considered statistically significant.

## Results

A total of 87 eyes from 49 PD patients and 73 eyes from 40 controls were analyzed after removing the acquisitions with visually identifiable motion artifacts, incomplete acquisitions or eyes presenting ocular exclusion criteria.

The demographics and clinical characteristics of participants are listed in Table 1. There were no statistically significant differences in age, but the proportion of females was larger in the control group. The mean disease duration was 7.1 ± 4.1 years (range 0.4 to 19.4 years), and the mean UPDRS motor score was 27.7 ± 7.7 (range, 9 to 54). The cognitive status was similar between PD and controls, but the proportion of subjects with MCI was larger in PD group. In PD patients, MoCA score presented a mild correlation with motor deficits (*r* = −0.292, *p* = 0.04). The frequency of well-controlled hypertension was comparable in both groups.

**TABLE 1:** Demographics and clinical characteristics of participants.

	**PD**	**Control**	***p*-value**
n	49	40	
Age (years)	64.6 (7.9)	62.1 (8.0)	0.2
Sex (female n, %)	16 (34.7%)	27 (67.5%)	<0.001
MoCA	24.4 (4.1)	25.7 (2.5)	0.3
MCI (n, %)	18 (36.7%)	6 (15%)	0.03
Hypertension (n, %)	12 (24.5%)	7 (17.5%)	0.59
Disease Duration (years)	7.1 (4.1)	–	
UPDRS I	2.0 (1.5)	–	
UPDRS II	10.8 (4.0)	–	
UPDRS III	27.7 (7.7)	–	
UPDRS IV	4.0 (2.9)	–	
LEDD (mg)	647.5 (364.6)	–	

*Categorical data are expressed as number and percentage, whereas quantitative data is expressed as mean (standard deviation). The proportion of participants with well-controlled or benign hypertension is provided for each group. LEDD, Levodopa Equivalent Daily Dose; MCI, Mild Cognitive Impairment; MoCA, Montreal Cognitive Assessment; n, sample size; PD, Parkinson’s disease; UPDRS, Unified Parkinson’s disease Rating Scale.*

PD patients were further divided into two groups: PD patients with MCI (PD-MCI) (*n* = 18) and PD patients with normal cognition (PD-NC) (*n* = 31). The mean age of PD-MCI was 67.1 ± 8.9 years and in PD-NC it was 63.1 ± 6.9 years (*p* = 0.11). Disease duration was comparable among both groups (PD-MCI 6.3 ± 4.4 vs. PD-NC 7.5 ± 4.0, *p* = 0.3). The proportion of females was also similar in both groups (PD-MCI 38.9% and PD-NC 32.2%, *p* = 0.9), as well as the proportion of patients with well-controlled hypertension (PD-MCI 16.6% and PD-NC 22.2%, *p* = 0.7).

### Comparison of Microvascular Parameters Between PD Patients and Controls

Comparing PD patients and controls, significant differences were found in FAZ area in SVC (*p* = 0.004) and DVC (*p* < 0.001) with a medium to large effect size, but not in FAZ circularity. After controlling for *a priori* confounders (i.e., age, sex, and hypertension), FAZ area remained significantly smaller in PD patients compared to controls in both SVC and DVC (estimate −0.1 μm, adjusted *p* = 0.004 in SVC and *p* = 0.014 in DVC) (Table 2).

**TABLE 2:** Foveal microvascular changes in PD.

		**PD**	**Control**	**Cohen’s *d***	**Univariate p-value**	**Multivariate *p*-value**
FAZ area (mm^2^)	SVC	0.669 ± 0.214	0.824 ± 0.292	0.61	**0.004**	**0.004**
	DVC	0.401 ± 0.181	0.544 ± 0.198	0.75	**<0.001**	**0.014**
FAZ circularity	SVC	0.187 ± 0.038	0.194 ± 0.029	0.21	0.280	0.686
	DVC	0.257 ± 0.045	0.271 ± 0.043	0.32	0.067	0.210
Lacunarity	SVC	6.0 ± 0.4	5.7 ± 0.4	0.75	**<0.001**	**<0.001**
	DVC	12.8 ± 0.7	9.8 ± 3.2	1.30	**<0.001**	**<0.001**
Fractal Dimension	SVC	1.42 ± 0.05	1.37 ± 0.09	0.69	**0.008**	**0.027**
	DVC	1.49 ± 0.04	1.47 ± 0.04	0.50	**0.030**	0.127
Perfusion Density	SVC	0.14 ± 0.04	0.11 ± 0.05	0.66	**0.009**	0.112
	DVC	0.22 ± 0.04	0.20 ± 0.05	0.44	**0.020**	**<0.001**
Skeleton Density (1/mm)	SVC	6.0 ± 1.9	4.8 ± 2.2	0.58	0.052	0.650
	DVC	8.6 ± 1.9	7.6 ± 1.8	0.54	**0.034**	**0.002**
Vessel Perimeter Index (1/mm)	SVC	17.1 ± 4.8	13.6 ± 5.6	0.67	**0.006**	0.210
	DVC	27.6 ± 5.2	23.6 ± 5.6	0.74	**0.003**	0.541
Vessel Diameter (μm)	SVC	23.3 ± 1.6	23.7 ± 2.1	0.21	0.160	0.056
	DVC	26.1 ± 2.1	25.8 ± 2.0	0.15	0.320	0.944

*Microvascular parameters are expressed as mean ± standard deviation for each group. Cohen’s d represents the effect size. p-values were obtained with GEE. Multivariate p-values are adjusted for age, sex, and hypertension. Significant results are highlighted in bold. Microvascular parameters without units are dimensionless. DVC, deep vascular complex; FAZ, foveal avascular zone; PD, Parkinson’s disease; SVC, superficial vascular complex.*

**FIGURE 2:**
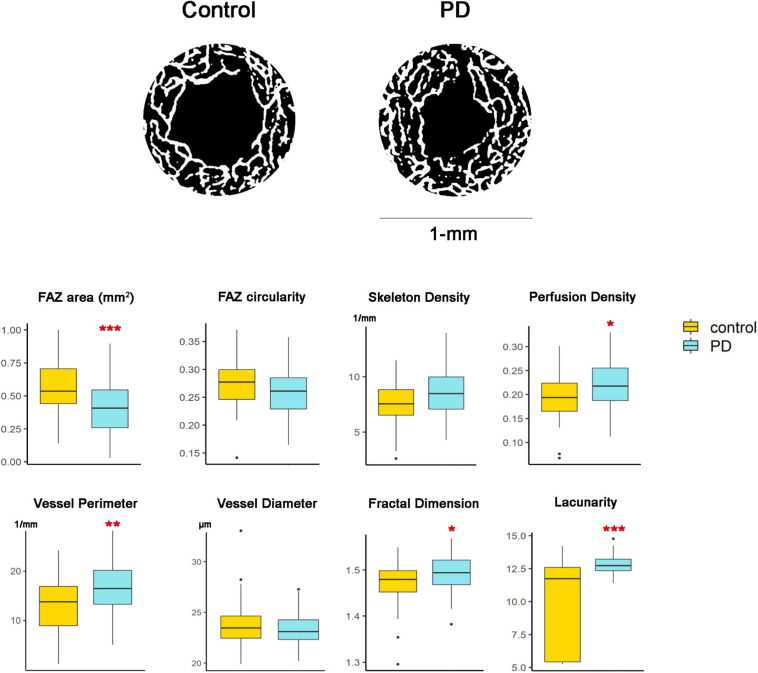
Representative images of the microvascularization in the foveal zone in controls and PD patients. Top panel figures show binarized images of the Deep Vascular Complex of the retina, centered in the centroid of the Foveal Avascular Zone (FAZ), in a circle with a radius of 500 μm. The scaling factor was 5.60 in both subjects, so the differences in FAZ size cannot be attributed to ocular biometric differences or magnification effects. Graphs correspond to the results in the Deep Vascular Complex. Significance levels of unadjusted GEE models are represented with an asterisk: *p < 0.05, ** *p* < 0.01, *** *p* < 0.001.

When analyzing differences in microvascular parameters between PD patients and controls, skeleton density, perfusion density and vessel perimeter of PD patients were increased in the foveal zone, with statistically significant differences compared to controls (Figure 2). Moreover, PD eyes showed increased FD and lacunarity of both complexes in the foveal zone. Adjusted GEE models showed that the SVC lacunarity and FD, and DVC lacunarity, skeleton density and perfusion density were significantly different between groups (Table 2), being the effect size particularly large for lacunarity. On the other hand, the parafoveal lacunarity in the retina of PD patients was significantly decreased in SVC and significantly increased in DVC (GEE, *p* < 0.001), but no differences were observed in the remaining parafoveal microvascular parameters.

### Retinal Thicknesses and Its Association With Microvascular Parameters

Multivariate GEE adjusted for age, sex and hypertension showed no significant differences in retinal thickness or its layers between PD patients and controls.

In the foveal zone, FAZ area was negatively associated with GCIPL and INL thickness in both PD patients and controls (*p* < 0.001), and no significant associations were found with ELM-IS/OS thickness in any group. These results suggest that the foveal microvasculature significantly contributes to OCT thickness measurement of inner retinal layers in normal and pathological conditions. In a similar fashion, in both groups, skeleton density, perfusion density, FD, and vessel perimeter of both plexuses were positively associated with the thickness of inner retinal layers (GCIPL and INL), but not with ELM-IS/OS.

However, a unique positive association was found in PD patients between microvascular parameters and OPL-ONL in the fovea. Concretely, FAZ areas in SVC and DVC were negatively associated, and skeleton density, perfusion density, and vessel perimeter of both plexuses were positively associated with OPL-ONL thickness, indicating that increased capillary bed in the fovea was related to OPL-ONL thickening. Also, foveal lacunarity of DVC was associated with GCIPL, INL and OPL-ONL thinning in PD patients, but not in controls.

In the parafovea of PD patients, a positive association was found between some microvascular parameters of the SVC, including skeleton density, perfusion density, FD and vessel perimeter with parafoveal GCIPL thickness (GEE, adjusted *p*-values: 0.014, 0.006, <0.001, and 0.013, respectively), but not with the thickness of the remaining retinal layer complexes. No such significant associations were found in control participants. None of the microvascular parameters of DVC were associated with retinal thicknesses in the parafovea.

### Retinal Parameters in PD Patients With Mild Cognitive Impairment

We also tested whether differences in microvascular parameters could be detected between PD patients with and without MCI. Some of such parameters tended to be lower in PD-MCI compared to PD-NC patients, like DVC lacunarity in the foveal zone or SVC skeleton density in the parafovea, but the differences did not reach statistical significance (Table 3). FAZ area was larger and FAZ circularity was decreased in PD-MCI patients, and both parameters were significantly different in SVC compared to PD-NC patients.

**TABLE 3:** Microvascular and thickness parameters in PD patients with and without MCI.

		**PD-MCI**	**PD-NC**	**Cohen’s *d***	**GEE *p*-value**
n		18	31		
FAZ area (mm^2^)	SVC	0.73 ± 0.20	0.63 ± 0.21	0.49	0.049
	DVC	0.43 ± 0.17	0.38 ± 0.18	0.29	–
FAZ circularity	SVC	0.17 ± 0.02	0.20 ± 0.04	0.95	0.001
	DVC	0.25 ± 0.05	0.26 ± 0.04	0.22	–
**Microvascular parameters**					
*Fovea*					
Fractal Dimension	SVC	1.40 ± 0.05	1.42 ± 0.05	0.40	–
	DVC	1.49 ± 0.04	1.50 ± 0.04	0.25	–
Lacunarity	SVC	6.02 ± 0.32	6.03 ± 0.48	0.02	–
	DVC	12.59 ± 0.63	12.91 ± 0.66	0.50	–
Skeleton Density (1/mm)	SVC	11.8 ± 1.5	11.7 ± 1.2	0.07	–
	DVC	12.0 ± 1.2	12.1 ± 1.3	0.08	–
Perfusion Density	SVC	0.28 ± 0.04	0.27 ± 0.03	0.28	–
	DVC	0.31 ± 0.02	0.32 ± 0.02	0.50	–
*Parafovea*					
Fractal Dimension	SVC	1.63 ± 0.02	1.63 ± 0.01	0	–
	DVC	1.69 ± 0.01	1.69 ± 0.01	0	–
Lacunarity	SVC	1.04 ± 0.01	1.04 ± 0.01	0	–
	DVC	10.98 ± 0.24	11.14 ± 0.24	0.67	–
Skeleton Density (1/mm)	SVC	11.4 ± 1.4	11.8 ± 1.3	0.30	–
	DVC	13.4 ± 1.2	13.5 ± 1.2	0.08	–
Perfusion Density	SVC	0.24 ± 0.02	0.24 ± 0.02	0.00	–
	DVC	0.35 ± 0.01	0.35 ± 0.01	0.00	–
**Thickness (μm)**					
*Fovea*					
Retina		277.1 ± 19.1	286.3 ± 18.2	0.49	0.045
GCIPL		36.0 ± 7.7	39.4 ± 7.9	0.44	–
INL		19.4 ± 6.1	21.0 ± 5.9	0.27	–
OPL-ONL		117.2 ± 13.7	123.3 ± 8.4	0.54	0.047
ELM-IS/OS		49.6 ± 6.2	49.1 ± 4.9	0.09	–
*Parafovea*					
Retina		339.4 ± 14.8	345.9 ± 13.3	0.46	–
GCIPL		92.0 ± 8.5	96.3 ± 7.2	0.55	0.039
INL		40.7 ± 2.8	40.7 ± 4.3	0	–
OPL-ONL		104.7 ± 8.1	106.1 ± 6.6	0.19	–
ELM-IS/OS		44.2 ± 3.5	44.0 ± 3.0	0.06	–

*Data are expressed as mean ± standard deviation for each group. Cohen’s d represents the effect size. p-values were obtained with univariate GEE. P-values are only provided for significant results. DVC, deep vascular complex; ELM-IS/OS, macular complex including external limiting membrane and inner and outer segments of photoreceptors; FAZ, foveal avascular zone; GCIPL, ganglion cell-inner plexiform complex; INL, inner nuclear layer; OPL-ONL, complex including the outer plexiform and outer nuclear layers; PD-MCI, Patients with Parkinson’s disease and Mild Cognitive Impairment; PD-NC, patients with Parkinson’s disease and normal cognition; SVC, superficial vascular complex.*

On the other hand, we observed that in PD-MCI retinal thickness was 6 μm lower in the parafovea and 9 μm lower in the foveal zone compared to PD-NC patients. Most of the parafoveal retinal thickness decrease in PD-MCI was accounted for changes in GCIPL (absolute difference of 4 μm). Contrarily, in the foveal zone, the GCIPL only accounted for a third part of the total retinal thinning (3 μm thinner), whereas foveal OPL-ONL thickness accounted for the rest (6 μm lower in PD-MCI vs. PD-NC, GEE, *p* = 0.047). However, none of these differences reached statistical significance after controlling for the effect of age, sex, and hypertension (Table 3).

Interestingly, we observed that parafoveal GCIPL thickness was significantly associated with parafoveal microvascular parameters in SVC, including skeleton density, perfusion density, fractal dimension, lacunarity and vessel perimeter, but only in PD-MCI and not in PD-NC.

### Association Between Microvascular Parameters and Clinical Outcomes

In PD patients, FAZ area and circularity of SVC were significantly associated with MoCA scores (GEE, *p* = 0.028, *p* = 0.036, respectively), but not with disease duration or UPDRS III scores. However, the relationship between superficial FAZ parameters and cognitive function lost significance when controlling for the effect of covariates. None of the remaining foveal or parafoveal microvascular parameters yielded significant associations with disease duration, motor impairment or cognitive outcomes.

### Diagnostic Accuracy of Macular Parameters

**FIGURE 3:**
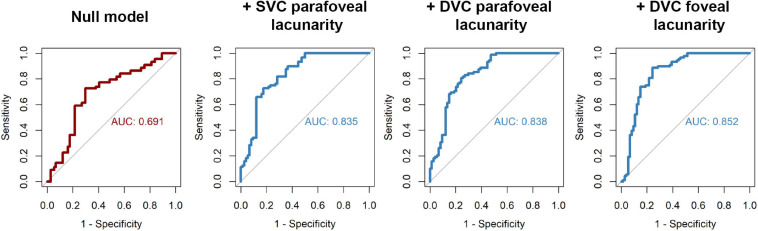
Receiver operating characteristic (ROC) curves for testing the diagnostic accuracy of microvascular parameters. Fitted values resulting from logistic regression were used as classifiers. ROC curve for the null model is shown in red, in which confounding demographical and clinical variables were used as independent factors, including age, sex, and hypertension. In blue, ROC curves of regression models that were significantly different from null (Wald test) after adding single microvascular parameters to the model. AUC, area under the curve; DVC, deep vascular complex; SVC, superficial vascular complex.

To test the diagnostic ability of OCT-A parameters alone or in combination with demographic, clinical, or retinal thickness variables, we fitted multivariable logistic GEE models and their predictive ability was tested in ROC curves. We first fitted the null model including age, sex, and hypertension as the *a priori* confounders. This yielded an area under the curve (AUC) of 0.691 (95% CI, 0.601 – 0.772). Then, we included single microvascular parameters that differed most between PD patients and controls, including FAZ area, foveal skeleton density, perfusion density and lacunarity (both plexuses), foveal FD in SVC and parafoveal lacunarity (both plexuses). Each model was then compared to the null with Wald test to test whether microvascular parameters significantly contributed to diagnostic accuracy. From this, we observed that 3 parameters significantly contributed to the model, providing excellent diagnostic accuracies (AUC > 0.8). These parameters were the lacunarity in DVC fovea (AUC = 0.852, 95% CI 0.79 – 0.92), lacunarity in DVC parafovea (AUC = 0.838, 95% CI 0.77 – 0.90) and lacunarity in SVC parafovea (AUC = 0.835, 95% CI 0.77 – 0.90) (Figure 3). Further adding retinal thicknesses of the parafovea or central macula or combining different microvascular parameters did not significantly improve the classification performance.

## Discussion

In the present study, we observed retinal capillary alterations in the central macula of PD patients. The area of the FAZ in both superficial and deep vascular plexuses was significantly smaller in PD patients compared to controls. In line with this finding, the perfusion and density of capillaries in the foveal zone was greater in PD patients, mainly in deep vascular plexus, suggesting an enlarged vascular bed surrounding FAZ. Moreover, fractal dimension and lacunarity of capillaries were greater in this region reflecting the increased vascular complexity and heterogeneity in PD fovea. Remodeling of foveal capillary bed was associated with increased OPL-ONL thickness in PD patients. Even though we failed to find differences in microvascular density or perfusion in the parafovea, parafoveal lacunarity significantly differed between patients and controls. Interestingly, parafoveal microvascular parameters on the superficial vascular complex were associated with GCIPL thickness in PD patients, but not in controls, and these associations were mainly driven by PD-MCI. Our results demonstrate that retinal microvascular alterations in PD are mainly restricted to the fovea, and that the parafoveal GCIPL atrophy in PD-MCI is associated with the superficial vascular supply.

Previous studies have explored retinal vascular alterations in PD. The first study analyzing retinal vascular changes in PD was conducted using fluorescein angiography (FA) (Miri et al., 2015). Although the resolution for fine capillary vessels of retinal FA is somewhat limited, these authors found a shrinking of FAZ in PD patients compared to controls, which is in line with the results of the current study using OCT-A images and improved algorithms to enhance the visualization of capillaries around the FAZ. Furthermore, we found that the decrease in FAZ area was accompanied by an increase in microvascular parameters in the foveal zone, like skeleton and perfusion density, fractal dimension or lacunarity. This contrasts with the results of Zou et al. who found less vessel length and perfusion in the central macula of PD patients and no changes in FAZ area (Zou et al., 2020), and with the results of Rascunà et al. who did not find vascular density changes in the foveal zone of early PD patients (Rascunà et al., 2020). Some of these differences might be attributed to smaller sample sizes, differences in disease stage of patients and study design flaws of previous studies.

The first study using OCT-A in PD was published in 2018, where Kwapong et al. (2018) nicely described a decrease in parafoveal microvascular density of superficial vascular complex in PD and its relationship with GCIPL thickness decrease. Similar results were reported by Shi et al. (2020). Nonetheless, none of these authors explored microvascular alterations in the foveal zone, where the fundamental microvascular alterations occur according to the present study. They did neither control for the inter-eye correlation in statistical analyses, possibly increasing the rate of false positive findings in the parafovea. In our study, we used GEE models to control for this effect, and did not find significant changes in the parafoveal skeleton or perfusion density. Nonetheless, part of their results coincides with ours, as we also found an association between the parafoveal microvascular density and GCIPL thickness, even after adjusting for confounding variables. Similarly, Rascunà et al. (2020), did not observe significant differences in the parafoveal microvascular density between early PD patients and controls but did find a correlation of inner retinal layer thickness and microvascular density. However, these correlations were mostly restricted to the foveal zone, and in the present study, we showed that such associations were not specific to PD, as they were also observed in controls. More recently, Robbins et al. (2021) used larger sample sizes, including 124 eyes of 69 PD patients and 248 eyes of 137 controls, concluding that retinal superficial capillary vessel density and perfusion density around the foveal zone are decreased in PD compared to age and sex-matched control participants, but no correlation analyses with retinal thickness were performed. Intriguingly, we observed that the association between parafoveal GCIPL thickness and microvascular parameters in PD was mainly driven by PD-MCI patients, whose parafoveal GCIPL was significantly reduced compared to PD-NC patients. As far as we know, this is the first study that classifies PD patients into subgroups in an attempt to unravel OCT-A differences between clinical endophenotypes. Even though few significant differences were found, PD-MCI tended to display larger FAZ areas and less FAZ circularity than PD-NC. Future studies with larger sample sizes might confirm this trend.

To date, few studies have assessed retinal vascular descriptors beyond density that can be useful for increasing the information obtainable from OCT-A images. Fractal dimension is a widely known parameter for describing shape or texture, and determines the complexity of an image. Two studies calculated FD of the retinal vasculature of PD patients finding contradictory results. Miri et al. (2015) did not find differences using FA, whereas Shi et al. (2020) reported decreased FD in the parafoveal superficial and deep vascular plexuses using OCT-A. This contrasts with our results, as we did not find differences in FD in the parafoveal region, but the FD within the foveal zone was significantly increased in PD patients. On the other hand, lacunarity is a feature descriptor that determines the heterogeneity of an image and complements FD. Lacunarity expresses patchiness or inhomogeneity of an image, and since it is not predicated on fractality, it may be particularly useful for characterizing the texture of retinal microvasculature. Indeed, in our study lacunarity was the parameter that differed most between patients and controls. The lacunarity within the foveal zone was significantly higher in PD patients compared to controls, even after adjusting for age, sex, and hypertension, suggesting that the distribution of capillaries was more heterogeneous in this region, with larger dispersion of gap sizes. The parafoveal lacunarity was greater in DVC in both PD patients and controls, as this plexus contains irregularly distributed vascular loops and vascular branches extending from a central seed point, notably increasing image heterogeneity (Hirano et al., 2018). Still, in the present work this parameter was also found to be significantly higher in PD patients compared to controls.

Moreover, lacunarity yielded the best diagnostic accuracy. Many efforts are being devoted to the identification and characterization of PD biomarkers. Notwithstanding the progress made so far, reliable biomarkers are still lacking. In this work, we showed that foveal lacunarity could be a promising biomarker for differentiating patients from controls, as a significantly greater AUC was achieved after adding this parameter to the null model that controlled for confounding variables like age, sex, and hypertension. In a previous study of Zou et al. (2020), similar AUCs were obtained after combining OCT-A and OCT parameters. However, these authors only accounted for capillary length and density, and no further microvascular parameters were considered. In our study, the combination of microvascular and structural modifications did not increase the classification accuracy, and lacunarity could be considered as a single imaging biomarker for discriminating patients from controls. The differences in microvascular imaging parameters between PD patients and controls, and the lack of association between these parameters with disease duration or severity, further support OCT-A as a useful diagnostic marker.

An important aspect to consider for the comparability of OCT-A studies is the critical contribution of technical differences among OCT-A devices. The definition of the delimiting boundaries of retinal vascular plexuses determines the tissue and associated vasculature that is represented in two-dimensional *en face* images. It is known that the location of these boundaries varies among OCT-A devices and limits the comparability of OCT-A studies (Li et al., 2018). So far, 2 OCT-A devices have been used to explore the vascular changes in PD, namely, AngioVue XR Avanti (Optovue Inc, Fremont, California, United States) and AngioPlexTM OCTA system (Cirrus; Zeiss, Dublin). In both devices, the intermediate capillary plexus (ICP) defined by Campbell et al. (2017) is considered to be part of SVC, whereas in Spectralis OCT-A the ICP pertains to DVC. Also, the axial resolution is similar among the 3 OCT-A devices (5 μm), but the lateral resolution of the OCT beam of Spectralis is much higher (5.7 μm) than the other two (15 μm). This is especially important considering that the average diameter of small capillaries is about 8 μm (Tan et al., 2012). Therefore, Spectralis OCT-A enables a more precise detection and more confident evaluation of vascular abnormalities at capillary level. As far as we know, this is the first study using Spectralis OCT-A in PD, and the mere use of this device could account for some of the contradictory results of our work compared to previous literature in PD.

The pathophysiological mechanisms driving retinal microvascular changes in PD are still unknown. In autopsy brains of PD patients, increased angiogenesis has been observed (Bradaric et al., 2012) with abnormally fragmented capillaries (Guan et al., 2013), increased expression of vascular endothelial growth (VEGF) receptors (Wada et al., 2006), and the formation of string vessels (Yang et al., 2015), supporting vascular events as a contributing factor to the PD pathophysiology. Concretely, string vessels are remnants of capillary vessels with no function in blood flow, and are triggered by factors that promote angiogenesis, like VEGF (Brown, 2010). As retinal and brain microvasculature share similarities, string vessel formation in the foveal zone could account for the current results. This hypothesis is partially supported by a recent in-vitro study that has demonstrated how degenerating retinal ganglion cells release VEGF to drive their own survival (Froger et al., 2020). Moreover, we observed that the relationship between microvascular parameters and OPL-ONL thickness in the fovea was exclusively present in PD patients, even though no significant thickness changes were detected between PD patients and controls in this area. It might be that the activation of VEGF receptor could not only promote pro-angiogenic effects, but also a microinflammation of the outer layers (Uemura et al., 2021). However, the mechanistic basis of microvascular retinal changes cannot be inferred from clinical studies.

Furthermore, the axons of retinal ganglion cells exit the eye to form the optic nerve, optic chiasm, and optic tract, which in turn synapse with other neurons in the lateral geniculate nucleus that extend through the optic radiations to the occipital lobe. Previous studies have observed morphometric abnormalities of the intracranial visual pathway structures in drug-naïve early PD patients, including decreased volume of chiasmatic area, and reduced white matter concentration and diffusivity in optic radiations (Arrigo et al., 2017). In future studies, it would be interesting to acknowledge not only whether the retinal microvascular alterations are associated to intracranial morphometric and functional changes, but also related to extracranial pathology, like pathology of carotid arteries.

Lastly, it is worth mentioning that the involvement of the eye in PD goes beyond the retina. In the last years, it has been demonstrated that PD patients show profound alterations of corneal innervation with decreased density of corneal subbasal nerve fibers and branches, and increased number of beadings (Arrigo et al., 2018; Ulusoy and Ulusoy, 2020). Probably, this denervation is responsible for the defective lacrimal reflex, and the subsequent dry eye signs and symptoms that are commonly reported in the literature (Biousse et al., 2004; Ekker et al., 2017).

One limitation of the current study was that the automatic segmentation of vascular plexuses near the fovea might not be completely accurate, as at this region the vascular plexuses converge into a single plexus around the FAZ, and DVC might be added to SVC in the fovea (Spaide and Curcio, 2017). However, we observed that microvascular changes in both plexuses were in line, but quantitative measurements should be interpreted with caution. Moreover, to find a tradeoff between the speed of acquisition and the resolution, the size of the field of view from the current OCT-A system was smaller than in previous studies. Nonetheless, we expected that macular changes in PD were predominantly restricted to the most central areas. Also, the cutoff for MCI was defined using MoCA scores, but future studies should rely on a comprehensive set of neuropsychological tests to refine the classification of PD patients. Several microvascular parameters were compared between PD patients and controls, and p-values were not corrected for multiple comparisons due to the exploratory nature of these analyses, and this remains a limitation of the current study. Finally, we did not account for all the potential confounding variables, such as smoking, intraocular pressure or axial length, although the effects of the latter were mitigated by scaling the OCT-A images. Future studies will benefit from using larger sample sizes to control for the effect of these factors in multivariable regression analyses.

In conclusion, the identification of biomarkers for PD is a mainstream in clinical research to forestall the progression of PD. Early retinal abnormalities in PD could permit a fast, non-invasive, and cost-effective imaging of surrogate markers. Our results support retinal vascular alterations detectable by OCT-A, mainly in the foveal zone, and as far as we know, our study is the first to describe changes in retinal vessel lacunarity in PD as a potential diagnostic biomarker of PD. Also, we indicate that PD patients with MCI might represent a clinical subtype in which retinal small vessel disease is related to retinal atrophy, although this working hypothesis needs to be tested in future studies. It is important to acknowledge the technical properties and limitations of each OCT-A device to ensure optimal interpretation of the obtained results in the clinical setting. The standardization of OCT-A vascular plexuses segmentation, the extraction and analysis of common vascular parameters and increasing the speed and resolution of acquisitions will enable a more precise description of retinal microvascular variations in PD. Without limiting the foregoing, vascular alterations in PD might need to be corroborated in postmortem retinas using histochemistry.

## Data Availability Statement

The raw data supporting the conclusions of this article will be made available by the authors, without undue reservation.

## Ethics Statement

The studies involving human participants were reviewed and approved by the CEIm de Euskadi (Comité de Ética de la Investigación con medicamentos). Osasun saila/Departamento de Salud. Eusko Jaurlaritza/Gobierno Vasco. C/Donostia-San Sebastián, 1 – 01010 Vitoria-Gasteiz. The patients/participants provided their written informed consent to participate in this study.

## Author Contributions

AM-G and IG conceptualized and designed the study. MB contributed to the implementation of the research. AE, UA, and DR-B processed imaging data. AM-G analyzed the data. ST-P collected imaging data and managed the project. JG-E recruited participants and collected neurological data. MA and RD collected cognitive data. AM-G wrote the manuscript with assistance from IG. All authors reviewed and contributed to the article and approved the submitted version.

## Conflict of Interest

The authors declare that the research was conducted in the absence of any commercial or financial relationships that could be construed as a potential conflict of interest.
